# Underwater coagulation using hybrid knife in peroral endoscopic myotomy for achalasia

**DOI:** 10.1055/a-2258-8371

**Published:** 2024-02-22

**Authors:** Antonio Capogreco, Roberto de Sire, Davide Massimi, Ludovico Alfarone, Roberta Maselli, Cesare Hassan, Alessandro Repici

**Affiliations:** 1 Gastroenterology, Endoscopy Unit, IRCCS Humanitas Research Hospital, Rozzano, Italy; 2 Department of Biomedical Sciences, Humanitas University, Pieve Emanuele, Italy

Achalasia is an idiopathic esophageal motility disorder, characterized by the insufficient relaxation of the lower esophageal sphincter and the absence of peristalsis in the esophageal body, leading to disabling symptoms, such as dysphagia, regurgitation, chest pain, and weight loss 1 . Until a few years ago, common interventional therapeutic approaches included botulinum toxin injection, endoscopic pneumatic balloon dilation, and laparoscopic Heller myotomy. Nevertheless, the first options have proved to be of only limited efficacy, while the surgical approach is highly invasive, costly, and burdened by potential severe complications and long recovery times 2 .

First described by Inoue et al., peroral endoscopic myotomy (POEM) has recently been introduced as a novel treatment option. This innovative technique consists of creating a submucosal tunnel and cutting the esophageal muscular fibers in a less invasive approach compared with surgery 3 . Owing to the excellent clinical success rates achieved in both the short and long term, with few adverse events, POEM has rapidly become one of the most common therapeutic approaches for esophageal achalasia 4 . However, despite these great results, it is still an expensive, long, difficult, and risky procedure.

Indeed, the creation of a submucosal tunnel is hampered by frequent intraprocedural bleeding that makes the procedure troublesome and time consuming, often requiring the use of costly hemostatic devices such as coagulation forceps. Such risk may also become clinically relevant as it may lead to major delayed bleeding that in turn may require hospitalization, blood transfusion, and re-treatment of the patient 5 .

No clear strategy for reducing the risk of intraprocedural and delayed bleeding has been put forward. Current practice consists of the preventive isolation and coagulation of the vessels identified during submucosal tunneling.

What we propose is a new technique for preventing the risk of intraprocedural bleeding and making POEM easier, quicker, safer, and cheaper. Indeed, we noticed that preventive underwater coagulation of the candidate vessels during submucosal tunneling with the HybridKnife (Erbe Elektromedizin GmbH, Tübingen, Germany), may seal the wall of the vessel, resulting in no bleeding when the vessel is subsequently cut under carbon dioxide insufflation ( Video 1 ). Such preventive coagulation is likely to be related to the conduction of the current under water as it focuses all the power on the interface between the vessel and the saline solution, allowing soft sealing of the vessel without cutting it ( Fig. 1 ).

A new technique for preventing the risk of intraprocedural bleeding during submucosal tunneling using underwater coagulation with hybrid knife in peroral endoscopic myotomy for achalasia.Video 1

**Fig. 1:**
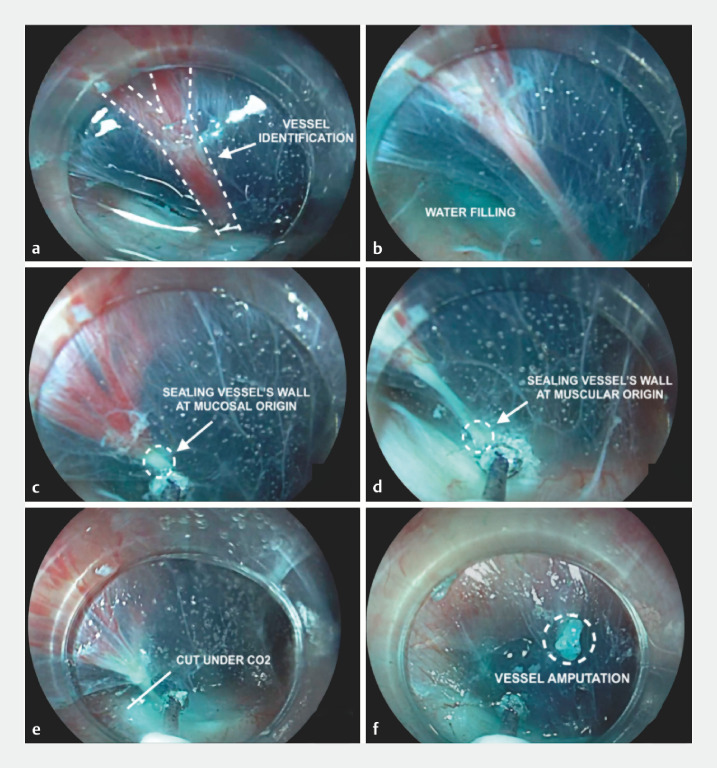
Phases of underwater coagulation using a HybridKnife (Erbe Elektromedizin GmbH, Tübingen, Germany) in peroral endoscopic myotomy for achalasia. **a** Vessel identification. **b** Filling with saline solution. **c** Sealing the wall of the vessel at the mucosal origin. **d** Sealing the wall of the vessel at the muscular origin. **e** Cutting the vessel under subsequent carbon dioxide insufflation. **f** Vessel amputation.

We suggest that the implementation of this novel approach in clinical practice may lead to an increase in safety, feasibility, and cost-effectiveness, reducing the procedural time, the rate of complications, and the need for coagulation forceps compared with the conventional preventive coagulation technique.

Endoscopy_UCTN_Code_TTT_1AO_2AD

## Notes

Endoscopy E-Videos https://eref.thieme.de/e-videos
*E-Videos* is an open access online section of the journal *Endoscopy* , reporting on interesting cases and new techniques in gastroenterological endoscopy. All papers include a high-quality video and are published with a Creative Commons CC-BY license. Endoscopy E-Videos qualify for HINARI discounts and waivers and eligibility is automatically checked during the submission process. We grant 100% waivers to articles whose corresponding authors are based in Group A countries and 50% waivers to those who are based in Group B countries as classified by Research4Life (see: https://www.research4life.org/access/eligibility/ ). This section has its own submission website at https://mc.manuscriptcentral.com/e-videos .
